# Gramicidin A in Asymmetric Lipid Membranes

**DOI:** 10.3390/biom14121642

**Published:** 2024-12-20

**Authors:** Oleg V. Kondrashov, Sergey A. Akimov

**Affiliations:** Frumkin Institute of Physical Chemistry and Electrochemistry, Russian Academy of Sciences, 31/4 Leninskiy Prospekt, 119071 Moscow, Russia

**Keywords:** gramicidin A, asymmetric lipid membrane, channel lifetime, equilibrium constant, theory of elasticity, membrane biophysics, lipid–protein interaction, lateral tension, intrinsic curvature

## Abstract

Gramicidin A is a natural antimicrobial peptide produced by *Bacillus brevis*. Its transmembrane dimer is a cation-selective ion channel. The channel is characterized by the average lifetime of the conducting state and the monomer–dimer equilibrium constant. Dimer formation is accompanied by deformations of the membrane. We theoretically studied how the asymmetry in lipid membrane monolayers influences the formation of the gramicidin A channel. We calculated how the asymmetry in the spontaneous curvature and/or lateral tension of lipid monolayers changes the channel lifetime and shifts the equilibrium constant of the dimerization/dissociation process. For the asymmetry expected to arise in plasma membranes of mammalian cells upon the addition of gramicidin A or its derivatives to the cell exterior, our model predicts a manifold increase in the average lifetime and equilibrium constant.

## 1. Introduction

**Figure 1:**
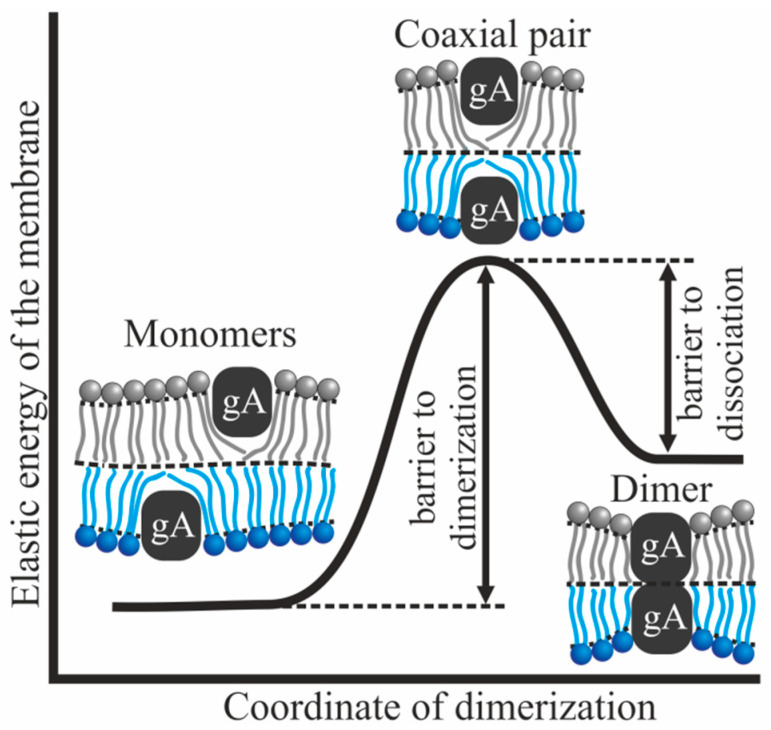
Configurations of two gA molecules located in opposing monolayers of the membrane: two monomers (left); conducting dimer (right); and coaxial pair (middle, top). The elastic energy of the membrane in these configurations is shown schematically. The states of two monomers and the conducting dimer are stable and metastable, respectively. These two configurations are in equilibrium with each other. The coaxial pair corresponds to the top of the energy barrier of the dimerization/dissociation process. The energy barrier of dimerization is the difference in the energies of the coaxial pair and two monomers; the energy barrier of dissociation is the difference in the energies of the coaxial pair and dimer. The only ion-conducting configuration is the dimer. Ionic conductance is harmful to cells as it leads to homeostasis violation.

Gramicidin A (gA) is a predominantly hydrophobic pentadecapeptide that forms a *β*^6.3^-helix when inserted into a lipid bilayer [1,2,3,4]. The transmembrane dimer, formed by two gA monomers located in opposite membrane monolayers, is a cation-selective ion channel [1,5]. The dimer is stabilized by six hydrogen bonds formed between valines located near the N-termini of the monomers [6]. The ion channel is characterized by the average lifetime of the conducting state (dimer) and the average number of dimers per unit membrane area at a given gA concentration [5,7,8]. The average number of dimers is directly related to the integral ion conductance of the membrane. The length of the transmembrane gA dimer is usually less than the thickness of a “typical” lipid bilayer (e.g., composed of dioleoylphosphatidylcholine, DOPC, or diphytanoylphosphatidylcholine, DPhPC). Therefore, it is believed that the dimer formation is accompanied by membrane deformations: dimer formation requires the compression of the adjacent part of the lipid bilayer [9,10]. Since the length of the gA monomer is less than the thickness of a “typical” lipid monolayer, the membrane is also deformed near the gA monomer [11,12,13]. Both the formation of gA dimer and its dissociation into two monomers occur via the same intermediate state of a coaxial pair, in which two gA monomers are located in opposite monolayers one on top of the other [11,12,13]. It is assumed that the state of the gA coaxial pair corresponds to the top of the energy barrier of the dimerization/dissociation reaction (Figure 1). The energy of membrane deformations contributes to the energy of all three gA states [11,12,13]. It has been experimentally shown that the channel characteristics do depend on the elastic parameters of the lipid membrane: thickness [14], spontaneous curvature of monolayers [10,15,16], and lateral tension [17,18].

Starting from the pioneering work by Huang [19], a large number of theoretical models have been developed, linking the elastic contribution to the energy of a particular gA state with the elastic parameters of the lipid bilayer [10,11,12,13,14,15,16,17,18,19,20,21]. The dependences of channel lifetime on spontaneous curvature [10,11,15,16,20,21], lateral tension [17,18,21], and membrane thickness [11,14,17,21] have been predicted and tested experimentally. However, both theoretical and experimental studies of gA have been carried out in symmetric bilayer systems, i.e., in which the physical properties of the monolayers are identical. Meanwhile, real biological membranes are usually significantly asymmetric in the lipid composition of monolayers. In particular, in most Gram-negative bacteria, the inner monolayer of the plasma membrane is enriched by phosphatidylethanolamine (PE), phosphatidylglycerol (PG), and cardiolipin, while the outer monolayer is enriched by lipopolysaccharides or glycosphingolipids [22]. In the plasma membranes of mammalian cells, the inner monolayer is enriched by PE, phosphatidylserine (PS), and phosphatidylinositol (PI) with predominantly unsaturated acyl chains, while the outer monolayer is enriched by phosphatidylcholine (PC), sphingomyelin (SM), and gangliosides with more saturated acyl chains [23,24,25,26].

The membrane asymmetry in lipid composition leads to differences in the physicochemical characteristics of the constituent lipid monolayers, primarily in their elastic parameters. Spontaneous curvature is one of the most sensitive elastic parameters to the type of polar lipid head and the length and unsaturation of acyl chains [27,28]. Generally, the spontaneous curvature of PE, PI, PG, and cholesterol is strongly negative, while that of PS, SM, PC, and gangliosides is about zero (slightly negative or positive) [27,28,29]. In addition to the spontaneous curvature, monolayers can have other different physical characteristics, such as bending moduli and lateral stretching moduli. If the lipid exchange between monolayers of a closed membrane, for example, a giant unilamellar vesicle (GUV), is slow enough, then different lateral tensions can arise in monolayers, up to complete asymmetry, i.e., when lateral tension arises in one monolayer, and lateral pressure approximately equal to it in absolute value arises in the opposite monolayer [30].

The generalization of theoretical models to the case of asymmetric membranes suggests the practical use of gA in biological systems. Gramicidin A has high bactericidal properties, but at working concentrations, it destroys erythrocytes. However, this peptide has good potential for modification. Charged analogs of gA [31,32] or gA with a reduced dimerization constant due to the replacement of N-terminal valines with other amino acids [12] can nevertheless be used in vivo for various purposes. In previous work [33,34], it was shown that [Glu1]gA, i.e., gA with an N-terminal valine replaced by Glu, can penetrate several lipid membranes in a model system and relieve the electrical potential on the internal membranes of mitochondria in cells. It has been shown in vivo that [Glu1]gA can alleviate the consequences of brain stroke in rats due to the fact that when the membrane potential is relieved, active oxygen species that damage neurons are not produced in mitochondria [33]. Such an application implies the possibility of gA transfer from one membrane monolayer to another, for example, from the outer monolayer of the plasma membrane to its inner monolayer. Obviously, the asymmetry of the lipid composition of the membrane monolayers should affect the flip–flop rate.

In this work, we studied how the asymmetry of the physical characteristics of monolayers affects the average lifetime of a solitary gA dimer and the monomer–dimer equilibrium constant. We focused on the relatively simple case of homogeneous monolayers, i.e., consisting of a single lipid component. The influence of heterogeneity and/or the presence of impurities on the average lifetime and equilibrium constant of gramicidin is described in detail in [35,36].

## 2. Methods

The average lifetime of gA dimer and the equilibrium constant of gA dimers and monomers are determined by the energies of three configurations of gA [11,12,13]: two isolated monomers located in different monolayers; a conducting dimer, which is a metastable configuration of two monomers; and a coaxial pair consisting of two gA monomers located one on top of the other in opposing monolayers (Figure 1). In all of these configurations, gA deforms the lipid membrane and deforms it differently. This results in energy barriers both to dimer formation and to its dissociation. The values of these barriers determine the average lifetime of the conducting state and the average number of dimers in the membrane.

In order to form the dimer, two gA monomers located in opposing monolayers must meet during their diffuse movement along the membrane surface, and form the coaxial pair by overcoming the energy barrier Δ*W_form_* = *W_pair_* − (*W_mon in_* + *W_mon out_*), where *W_pair_* is the membrane deformation energy in the coaxial pair state and *W_mon in_* and *W_mon out_* are the membrane deformation energies induced by single monomer in the inner and outer monolayers, respectively. Having reached the coaxial pair state, the top of the potential barrier, gA monomers can form the conducting dimer or escape back to isolated monomers. Thus, the rate constant *K_dim_* of the dimerization reaction can be written as:

(1) $$K_{dim}=K_{dim\;0}\exp\left[-\frac{\Delta W_{form}}{k_{B}T}\right]=K_{dim\;0}\exp\left[-\frac{W_{pair}-\left(W_{mon\;in}+W_{mon\;out}\right)}{k_{B}T}\right],$$

where *K_dim_*_0_ is the pre-factor depending mostly on the diffusion rate and on the membrane-mediated lateral interaction of gA species in the case of large concentrations [12,35]; *k_B_* is the Boltzmann constant; *T* is the absolute temperature. In the following, we consider the case of low concentrations of gA, when the lateral interaction between gA particles can be neglected and the value of *K_dim_*_0_ can be considered constant.

When a dimer dissociates into two monomers, it transforms into a coaxial pair. Thus, the average lifetime *τ* of the dimer can be written as:

(2) $$\tau =\frac{1}{\nu }\exp\left[-\frac{{W^{\prime }}_{dim}-W_{pair}}{k_{B}T}\right],$$

where *ν* is the characteristic frequency of attempts of dimer dissociation; *W*′*_dim_* is the energy in the dimer state taking into account the energy of stabilizing hydrogen bonds. Since the value of 1/*τ* is the rate constant of decay of the conducting state, then for the equilibrium constant of the dimerization/dissociation reaction *K*, we have:

(3) $$K\equiv \frac{C_{d}}{C_{mon\;in}C_{mon\;out}}=K_{dim}\tau =K^{\prime }\exp\left[-\frac{W_{dim}-\left(W_{mon\;in}+W_{mon\;out}\right)}{k_{B}T}\right],$$

where *C_d_*, *C_mon in_*, and *C_mon out_* are surface concentrations of dimers, monomers in the inner monolayer, and monomers in the outer monolayer, respectively; *K*′ is some constant pre-factor and *W_dim_* is the energy in the dimer state without accounting for the contribution of hydrogen bonds. From expressions (2) and (3), it follows that in order to calculate the relative change in the average lifetime of the dimer or the equilibrium constant in the asymmetric membrane, it is necessary to calculate how the energies of the above-described gA configurations change (Figure 1). In this case, all contributions to the energy due to chemical interactions, for example, the energy of hydrogen bonds of the dimer, will not contribute to the final expressions for the relative changes of the average lifetime and equilibrium constant.

To calculate the energies of gA configurations for arbitrary elastic parameters of lipid monolayers, we used the functional elastic deformation energy of the membrane developed by Hamm-Kozlov [37] and further generalized in [11,12,13]. In this approach, deformations of the lipid monolayer are characterized by the shape of its neutral surface *H*, the field of unit vectors **n**, called directors, which correspond to the average direction of the longitudinal axes of anisotropic lipid molecules, and the shape of the monolayer interface *M*. The deformation energy of the bilayer is the sum of the deformation energies of two monolayers. We consider the outer monolayer as the upper one, and denote the variables related to it by the index “*u*” and the inner monolayer as the lower one (index “*l*”). The deformations are considered small, and the energy is calculated in the second order with respect to them. The bulk modulus of lipid membranes is very high, about ~10^10^ J/m^3^ [38]. For this reason, the hydrophobic part of lipid monolayers can be considered volumetrically incompressible. Within the required accuracy, the condition of the local volumetric incompressibility can be written as [11,12,13,37]:

(4) $$\alpha_{u,l}=\frac{1}{h_{u,l}}\left[\pm M+h_{u,l}\mp H_{u,l}-\frac{h_{u,l}^{2}}{2}\mathrm{div}\left(n_{u,l}\right)\right],$$

where *α* is the relative stretching; *h* is the thickness of the hydrophobic part of the lipid monolayer. The energy of elastic deformation can be written as [11,12,13]:

(5) $$\begin{array}{l} W=\int dS_{u}\left(\frac{B}{2}{\left(\mathrm{div}n_{u}+J_{0u}\right)}^{2}-\frac{B}{2}J_{0u}^{2}+\frac{K_{t}}{2}{\left(n_{u}-grad\;H_{u}\right)}^{2}+\frac{\sigma_{u}}{2}{\left(grad\;H_{u}\right)}^{2}\right.\\ \left.+\frac{K_{a}}{2h^{2}}{\left(h-\frac{h^{2}}{2}\mathrm{div}n_{u}+M-H_{u}\right)}^{2}+K_{G}K_{u}\right)\\ +\int dS_{l}\left(\frac{B}{2}{\left(\mathrm{div}n_{l}+J_{0l}\right)}^{2}-\frac{B}{2}J_{0l}^{2}+\frac{K_{t}}{2}{\left(n_{l}+grad\;H_{l}\right)}^{2}+\frac{\sigma_{d}}{2}{\left(grad\;H_{l}\right)}^{2}\right.\\ \left.+\frac{K_{a}}{2h^{2}}{\left(h-\frac{h^{2}}{2}\mathrm{div}n_{l}-M+H_{l}\right)}^{2}+K_{G}K_{l}\right), \end{array}$$

where *B* is the splay modulus; *J*_0_ is the spontaneous curvature of lipid monolayer; *K_t_* is the tilt modulus; *σ* is the lateral tension; *K_a_* is the modulus of lateral stretching; *K_G_* is the Gaussian modulus; and *K_u_*_,*l*_ is the Gaussian splay. The integration is performed over the surfaces of upper and lower monolayers. The deformation of relative stretching was substituted from the condition of local volumetric incompressibility, as seen in Equation (4). The deformations in (5) are assumed to be small, so **n***_u_* = (*n_ux_*, *n_uy_*, −1), **n***_l_* = (*n_lx_*, *n_ly_*, +1), where *n_x_* and *n_y_* are corresponding projections of the director in a Cartesian coordinate system, of which the *xy* plane coincides with the plane of the unperturbed membrane. We further focus on how the energy of gA configurations change if there are asymmetric tension *σ_u_* ≠ *σ_l_* and/or spontaneous curvature *J*_0*u*_ ≠ *J*_0*l*_; all other parameters are assumed to coincide in the upper and lower monolayers.

The minimization of the energy functional (5) requires appropriate boundary conditions. There are no deformations far from gA, so we can write:

(6) $$\begin{array}{l} H_{u}\left(\infty \right)=h,\;H_{l}\left(\infty \right)=-h,\;M\left(\infty \right)=0,\\ n_{u}\left(\infty \right)=(0,0,-1),\;n_{l}\left(\infty \right)=(0,0,+1). \end{array}$$

Each configuration of gA imposes its own boundary conditions, which were described in detail in previous works [11,12,13]. Here, we describe them briefly. First of all, all three gA configurations are cylindrically symmetrical. The boundary of gA at the neutral surface of the corresponding monolayer is assumed to be a circle Γ of radius *r*_0_. The gA monomer imposes boundary conditions only on director projections onto the plane of the unperturbed membrane:

(7) $$n_{n}\left(\Gamma \right)=-\frac{h-h_{p}}{\sqrt{{\left(h-h_{p}\right)}^{2}+h_{p}^{2}}}=n_{0},\;n_{t}\left(\Gamma \right)=0,$$

where *n_n_* and *n_t_* are normal and tangential to Γ projections of the director **n**, and *h_p_* is the length of the hydrophobic part of the gA monomer. The gA monomer imposes boundary conditions (7) only on the director of the monolayer where the gA monomer is located. The gA pair imposes two boundary conditions similar to (7) for directors in both monolayers. The gA dimer sets the membrane thickness at the contour Γ:

(8) $$H_{u}\left(\Gamma \right)-H_{l}\left(\Gamma \right)=2h_{p}.$$

To find the minimum of functional (5) under boundary conditions (6)–(8), we derive the Euler–Lagrange equations in the cylindrical coordinate system *Orz*. We do not present them here because they are too bulky; they were obtained explicitly in previous work [11]. Euler–Lagrange equations are linear, and they were solved analytically. For the difference in director projections in the upper and lower monolayers *d*(*r*) = *n_u_*(*r*) − *n_l_*(*r*), the general solution of Euler–Lagrange equations has the form:

(9) $$\begin{array}{l} d\left(r\right)=c_{0}\frac{1}{r}+c_{1}r+c_{2}J_{1}\left(p_{1}r\right)+c_{3}J_{1}\left(p_{2}r\right)+c_{4}J_{1}\left(p_{3}r\right)\\ +c_{5}Y_{1}\left(p_{1}r\right)+c_{6}Y_{1}\left(p_{2}r\right)+c_{7}Y_{1}\left(p_{3}r\right), \end{array}$$

where *c*_0_, *c*_1_, …, *c*_7_ are constant complex coefficients that should be determined from the boundary conditions; *J*_1_ and *Y*_1_ are the corresponding Bessel functions of the first order; *p*_1_, *p*_2_, and *p*_3_ are inverse characteristic lengths of deformations determined by the elastic parameters only. To determine a part of coefficients *c*_0_, *c*_1_, …, *c*_7_ we used the boundary conditions from Equations (6)–(8). The remaining coefficients were determined by the direct minimization of (5) after the substitution of the general solution for deformations in a form similar to (9).

We calculated the energies of gA configurations for different values of spontaneous curvature and lateral tension in the upper and lower monolayers. Of note, the energy contribution from the spontaneous curvature can be explicitly integrated from the functional Equation (5). For the gA monomer located in the inner (outer) monolayer, the corresponding term yields:

(10) $$\begin{array}{l} W_{mon\;in\;(out)}=W_{mon\;in\;(out)0}+\int_{r_{0}}^{+\infty }dS_{u(l)}\left(2\frac{B}{2}J_{0u(l)}\mathrm{div}n_{u(l)}\right)\\ =W_{mon\;in\;(out)0}+\int_{r_{0}}^{+\infty }2\pi rBJ_{0u(l)}\left({n^{\prime }}_{u(l)}+\frac{n_{u(l)}}{r}\right)dr=W_{mon\;in\;(out)}-2\pi r_{0}BJ_{0u(l)}n_{0}, \end{array}$$

where $W_{mon\;in\;(out)0}$ is a constant term independent of the spontaneous curvature. From Equation (10), it follows that the energy of deformations induced by the gA monomer depends linearly on the spontaneous curvature of the monolayer where the monomer resides.

To obtain quantitative results, we used the following values of the elastic parameters (per monolayer): splay modulus *B* = 10 *k_B_T* (*k_B_T* ≈ 4 × 10^−21^ J) [39]; tilt modulus *K_t_* = 40 mN/m [37,40]; lateral stretching modulus *K_a_* = 133 mN/m [39]; Gaussian splay modulus *K_G_* = −0.3*B* [41]; thickness of the hydrophobic part of the monolayer *h* = 1.45 nm [11,12,13]; the diameter of gA monomer 2*r*_0_ = 2 nm; the length of gA dimer 2*h_p_* = 1.5 nm [11,12,13]. We considered the following values of spontaneous curvatures of the upper and lower monolayers: *J*_0_ = −0.3, −0.2, −0.1, 0 nm^−1^. The lateral tension and pressure in the monolayers were varied from −3 mN/m to +3 mN/m with the imposed condition of positive total lateral tension of the membrane, i.e., *σ_u_* + *σ_l_* > 0, that is, the condition of membrane mechanical stability.

## 3. Results

**Figure 2:**
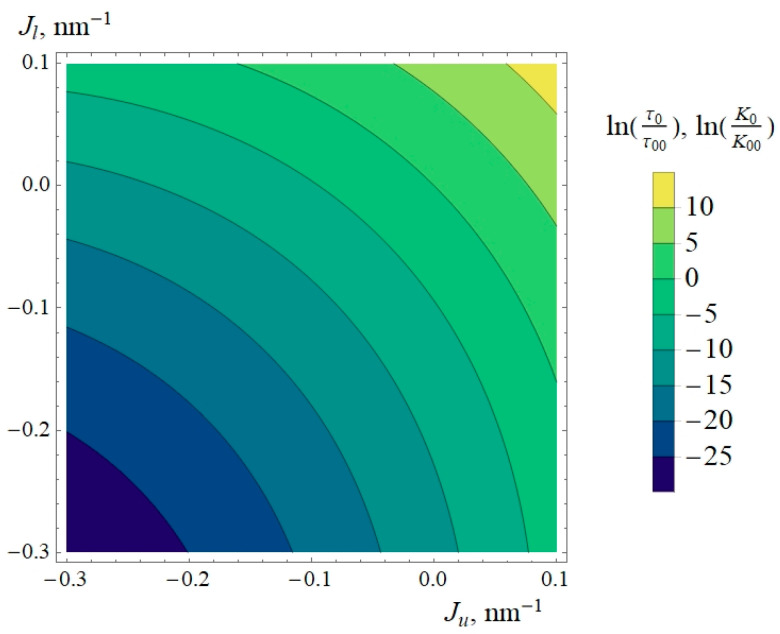
Dependences of logarithms of normalized dimer lifetime *τ*_0_/*τ*_00_ and normalized equilibrium constant *K*_0_/*K*_00_ at almost zero lateral tensions, *σ_u_* = *σ_l_* ≈ 0 (corresponding to plasma membranes of cells or deflated GUVs) on spontaneous curvatures of the outer (*J_u_*) and inner (*J_l_*) monolayers. Larger *K*_0_ corresponds to larger equilibrium number of dimers and higher integral conductance of the membrane.

Using Equations (2) and (3), we calculated the gA dimer average lifetime and monomer–dimer equilibrium constant. To compare the results obtained for different sets of the elastic parameters, we assumed that the frequency *ν* and constant $K^{\prime }$ are the same for different lipids or their dependences on spontaneous curvature are weak and we can neglect them in comparison with exponential dependences in Equations (2) and (3). As the reference point, we used the limit (*σ_u_*, *σ_l_*) → (0, 0): the corresponding limits of lifetime *τ* and equilibrium constant *K* are denoted as *τ*_0_ and *K*_0_. It should be noted that the elastic energy functional Equation (5) is stable only when *σ_u_* + *σ_l_* > 0, and values *σ_u_* = *σ_l_* = 0 do not correspond to any stable membrane. However, if (*σ_u_* + *σ_l_*) is positive, the energies of all states of gA can be determined and the limit (*σ_u_*, *σ_l_*) → (0, 0) exists and corresponds to the membrane with infinitely small tension, for example, a deflated vesicle. To compare the lifetime *τ*_0_ and equilibrium constant *K*_0_, we introduce the reference values *τ*_00_ = *τ*_0_(*J_u_* = 0, *J_l_* = 0) and *K*_00_ = *K*_0_(*J_u_* = 0, *J_l_* = 0). Earlier, we showed that the dependences of the gA pair energy *W_pair_* and the sum of energies of two isolated monomers in different monomers (*W_mon in_* + *W_mon out_*) on spontaneous curvature are the same: they are linear with a coinciding slope coefficient [11]. For this reason, the dependences of *τ*_0_ and *K*_0_ on spontaneous curvature should coincide as well, and they can be plotted on the same graph (Figure 2).

From Figure 2, it is clear that the gA dimer lifetime *τ*_0_ and equilibrium constant *K*_0_ are highly sensitive to the spontaneous curvature of lipid monolayers. This result complements and generalizes the result of the previous work [11], where only the membranes composed of symmetrical monolayers were considered.

**Figure 3:**
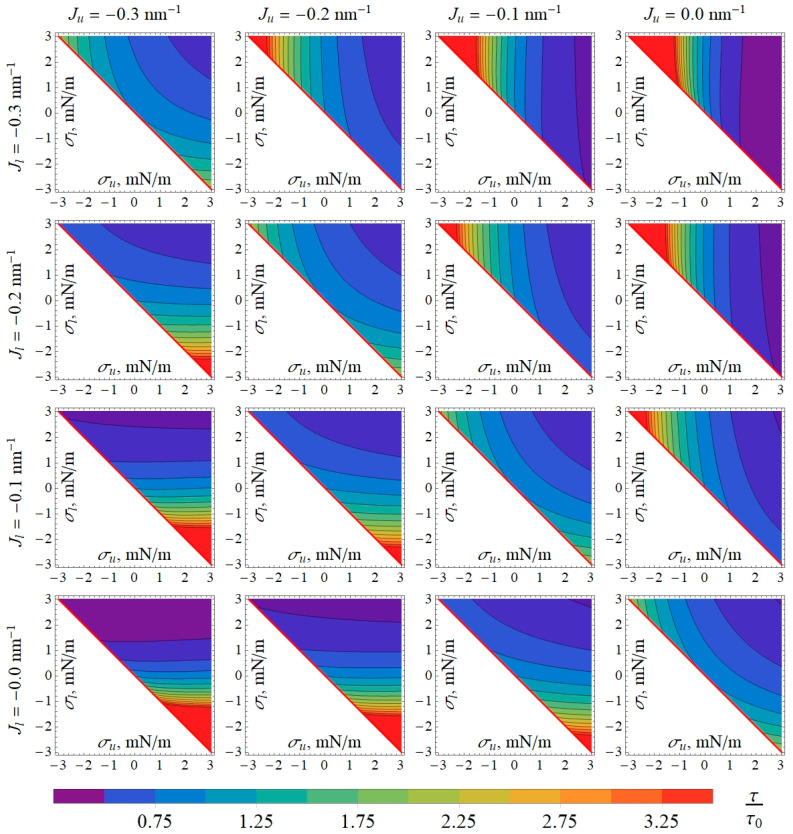
Dependence of normalized gA dimer lifetime *τ*/*τ*_0_ on lateral tensions in the outer (*σ_u_*) and inner (*σ_l_*) monolayers for different values of spontaneous curvature of the outer (*J_u_*) and inner (*J_l_*) monolayers. The values of *τ*_0_ were obtained as the limit of *τ* when (*σ_u_*, *σ_l_*) → (0, 0), corresponding to plasma membranes of cells or deflated GUVs. In white triangles in left-lower corners of the plots, *σ_u_* + *σ_l_* < 0, and the membrane is mechanically unstable. Thus, only right-upper halves of the plots are displayed.

**Figure 4:**
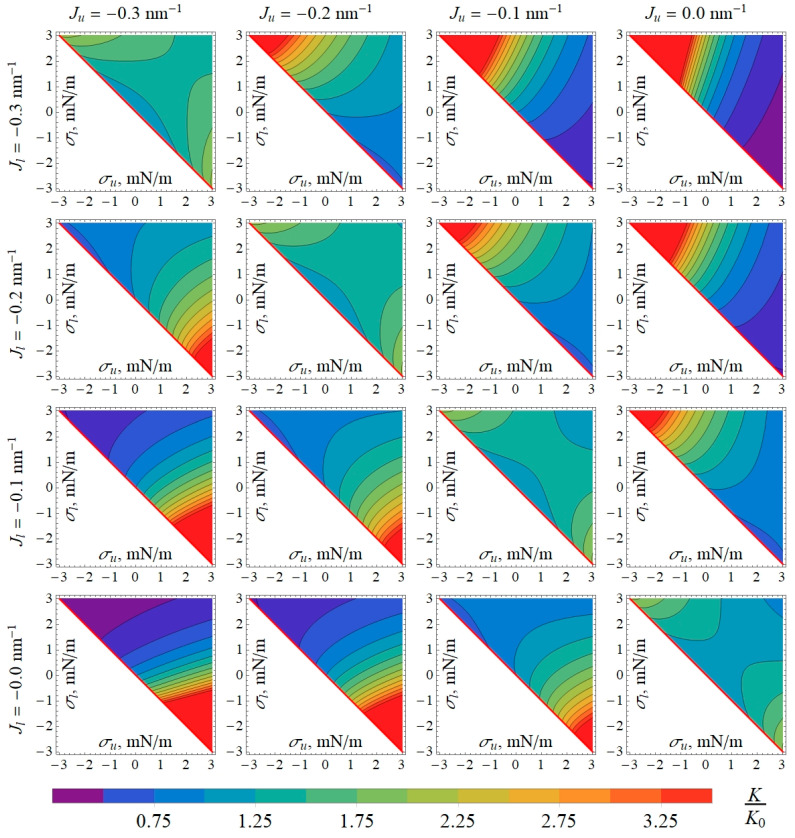
Dependence of normalized gA dimer–monomer equilibrium constant *K*/*K*_0_ on lateral tensions in the outer (*σ_u_*) and inner (*σ_l_*) monolayers for different values of spontaneous curvature of the outer (*J_u_*) and inner (*J_l_*) monolayers. The values of *K*_0_ were obtained as the limit of *K* when (*σ_u_*, *σ_l_*) → (0, 0), corresponding to plasma membranes of cells or deflated GUVs. In white triangles in left-lower corners of the plots, *σ_u_* + *σ_l_* < 0, and the membrane is mechanically unstable. Thus, only right-upper halves of the plots are displayed.

To investigate how the lateral tension influences the characteristics of the gA system, we calculated the gA dimer lifetime *τ* (Figure 3) and equilibrium constant *K* (Figure 4) at different lateral tensions of membrane monolayers. From Figure 3 and Figure 4, one can see that the lateral tension also regulates the gA lifetime and equilibrium. We should note that the asymmetric lateral tension can either increase or decrease *τ* and *K*, and the effect depends strongly on the spontaneous curvatures of the monolayers.

As the reference points, one can use the values of *τ* determined in [42] on model bilayer lipid membranes with lateral tension of about *σ_u_* = *σ_l_* = 0.5 mN/m: *τ* = 3.6 ± 1.1 s for the DOPC membrane (*J_u_* = *J_l_* = −0.091 nm^−1^ [28]), and *τ* = 4.5 ± 1.5 s for the DPhPC membrane (*J_u_* = *J_l_* = −0.097 nm^−1^ [42]). In [42], membranes were formed by the Montal–Muller technique without decane used as a solvent. For this reason, the presented values of *τ* differ from those determined on Muller–Rudin decane- or hexadecane-containing model membranes [14,15,18].

## 4. Discussion

Here, we developed a theoretical model for the calculation of the energy of elastic deformations induced by gA monomers, coaxial pairs, and dimers in lipid membranes that are asymmetric with respect to the lipid composition (spontaneous curvature) and lateral tension of lipid monolayers. The important characteristics of gA channels—the average lifetime and monomer–dimer equilibrium constant—depend exponentially on the calculated energies (Equations (2) and (3)). The asymmetry with respect to the composition of lipid monolayers is typical for biological membranes, particularly the plasma membranes of mammalian cells [24,25,26].

In typical mammalian plasma membranes, the outer monolayer is generally composed of more saturated lipids than the inner monolayer [24,25,26]. In addition, phosphatidylethanolamines with small polar heads are preferentially located in the inner monolayer. Overall, this means that the spontaneous curvature of the inner monolayer should be more negative than that of the outer monolayer. Since the length of the gA monomer is smaller than the thickness of the hydrophobic part of a typical lipid monolayer, its effective molecular shape when inserted into the monolayer is an inverted cone with its base directed toward the aqueous phase. This is formally reflected in the boundary condition in Equation (7): the gA monomer imposes a negative radial projection of the boundary director. The energy of deformations induced by the gA monomer is thus reduced in a lipid monolayer with negative spontaneous curvature and increased in a monolayer with positive spontaneous curvature. For the plasma membrane, this means that it is energetically more favorable for gA monomers to be seated in the inner monolayer since its spontaneous curvature is more negative than that of the outer monolayer. If gA or its derivatives are added from the outside of the cell, the asymmetry of the plasma membrane in the spontaneous curvature of its constituent monolayers should thus lead to the accumulation of gA monomers in the inner monolayer. This formally follows from Equation (10).

The predictions of the developed theoretical model can be quantitatively tested in compositionally asymmetric model membranes. Recently, methods of preparation of compositionally asymmetric large unilamellar vesicles (LUVs) have been developed (see, for example, [43]). The asymmetry is achieved with the use of the lipid-exchanging substance methyl-*β*-cyclodextrin (M*β*CD). M*β*CD cannot penetrate the membrane and thus modifies the lipid composition of the outer monolayer only. It is quite tricky to study the ion conductance of gA on LUVs. Asymmetric planar lipid bilayers formed by the Montal–Mueller technique [44] or its modification [42,45] are better suited to electrophysiological measurements. The model predictions of the average lifetime and number of dimers of gA can be tested in such model systems.

The asymmetry of the lipid membrane with respect to the lateral tension in its monolayers may arise when an amphiphilic substance (for example, gA) is added to either the outer or inner space of the closed membrane like GUVs. In this case, the incorporation of an amphiphilic substance into the accessible monolayer leads to an increase in the monolayer area. As the area of two monolayers of the membrane must almost coincide, the opposing monolayer would have to stretch laterally, while the accessible monolayer would have to laterally compress [30]. If the area introduced by the amphiphilic substance to its accessible monolayer is *S_p_*, then in equilibrium, neglecting the effects of thermal fluctuations of the membrane shape, the lipid of the accessible (e.g., outer) monolayer would be compressed by *S_p_*/2, while the lipid in the opposing (inner) monolayer would be stretched by *S_p_*/2; the overall area of the initially tensionless closed membrane would increase by *S_p_*/2. Such a change in the area of monolayers would lead to the generation of lateral pressure in the outer monolayers and equal (by absolute value) lateral tension in the inner monolayer. If thermal fluctuations of the membrane shape are taken into account, the result will be qualitatively the same (manuscript in preparation). In the case of the membrane that is symmetric with respect to the lipid composition (and spontaneous curvature) of its monolayers, for example, the GUV membrane, the asymmetry in lateral pressure/tension arising upon the addition of gA to the GUV exterior would increase both the average lifetime (Figure 3) and the equilibrium constant (Figure 4) by about 1.5–1.8 times if the lateral pressure/tension are in the order of ±3 mN/m. This effect is amplified up to >3.5 times when the spontaneous curvature of the outer monolayer is more positive than the spontaneous curvature of the inner monolayer (Figure 3 and Figure 4); this situation is characteristic of plasma membranes of mammalian cells [24,25,26].

To conclude, we considered the elastic energy of deformations induced by gA monomers, dimers, and coaxial pairs in the membranes asymmetric with respect to spontaneous curvature and lateral tension of lipid monolayers. Both types of asymmetry are predicted to contribute to the average lifetime of gA channels and the monomer–dimer equilibrium constant. For the asymmetry expected to arise in the plasma membranes of mammalian cells upon the addition of gA or its derivatives to the cell exterior, our model predicts a manifold increase in the average lifetime and equilibrium constant.

## Data Availability

The original contributions presented in this study are included in the article. Further inquiries can be directed to the corresponding authors.
